# Improving MASLD Risk Stratification in Young Adults with Cardiometabolic Risk Factors and Insulin Resistance Assessment

**DOI:** 10.1210/jendso/bvaf209

**Published:** 2025-12-16

**Authors:** Anu Sharma, Eddison Godinez Leiva, Srilaxmi Kalavalapalli, Andrea Ortiz Rocha, Nathaly Cuervo-Pardo, Jens Rosenberg, Pierre Bedossa, Romina Lomonaco, Fernando Bril, Diana Barb, Kenneth Cusi

**Affiliations:** Division of Endocrinology, Diabetes and Metabolism, University of Florida College of Medicine, Gainesville, FL 32610, USA; Division of Endocrinology, Diabetes and Metabolism, University of Florida College of Medicine, Gainesville, FL 32610, USA; Division of Endocrinology, Diabetes and Metabolism, University of Florida College of Medicine, Gainesville, FL 32610, USA; Division of Endocrinology, Diabetes and Metabolism, University of Florida College of Medicine, Gainesville, FL 32610, USA; Division of Endocrinology, Diabetes and Metabolism, University of Florida College of Medicine, Gainesville, FL 32610, USA; Advanced Magnetic Resonance Imaging and Spectroscopy Facility, McKnight Brain Institute, University of Florida, Gainesville, FL 32610, USA; Department of Pathology, Hôspital Beaujon, AP-HP Nord, Clichy 92110, France; Division of Endocrinology, Diabetes and Metabolism, University of Florida College of Medicine, Gainesville, FL 32610, USA; Division of Endocrinology, Diabetes and Metabolism, University of Alabama, Birmingham, AL 35294, USA; Division of Endocrinology, Diabetes and Metabolism, University of Florida College of Medicine, Gainesville, FL 32610, USA; Division of Endocrinology, Diabetes and Metabolism, University of Florida College of Medicine, Gainesville, FL 32610, USA

**Keywords:** young adults, MASLD/NAFLD, diabetes mellitus type 2, obesity, cardiometabolic risk factors

## Abstract

**Context:**

The fibrosis-4 index (FIB-4) index is recommended to identify adults with metabolic dysfunction-associated steatotic liver disease (MASLD) and clinically significant fibrosis (moderate to advanced fibrosis or ≥F2). However, it is less reliable in young adults (age <45 years).

**Objective:**

The aim was to assess whether cardiometabolic risk factors [CMRFs: type 2 diabetes (T2D), hypertension, obesity] or insulin resistance (IR) improved MASLD fibrosis risk stratification in young adults.

**Methods:**

Adults with/without T2D and no history of MASLD attending outpatient clinics underwent screening with vibration-controlled transient elastography for ≥F2 (liver stiffness measurement ≥ 8.0 kPa). Magnetic resonance elastography and/or liver biopsy were performed if indicated for diagnosis confirmation.

**Results:**

Of the 964 adults, 25% were young adults and 75% were 45 to 64 years, with the prevalence of ≥F2: 7% vs 9% (*P* = .29), respectively. In young adults, clinically significant fibrosis was unlikely in those without homeostatic model assessment of insulin resistance (HOMA-IR) or CMRFs [negative predictive value (NPV) 97-100; 95% confidence interval 94-100]. Performance of FIB-4 ≥ 1.3 had low sensitivity (15%) and positive predictive value (25%) but good specificity (97%) and NPV (95%), whereas having 3 CMRFs alone performed better (sensitivity 75%, specificity 71%). Adding FIB-4 ≥ 1.3 to CMRFs worsened sensitivity (8%) while improving specificity (100%). Adding the HOMA-IR to CMRFs improved the sensitivity (75% to 78%) and specificity (75% to 81%) of CMRFs alone. Adding 2 CMRFs to the FIB-4 in the older age group improved both sensitivity and specificity of the FIB-4.

**Conclusion:**

In young adults, the absence of CMRFs or IR makes clinically significant fibrosis unlikely. Measuring IR improved risk stratification in young adults with CMRFs. Using CMRFs with IR may improve the detection of clinically significant fibrosis in young adults.

The alarming rise in cardiometabolic risk factors (CMRFs) in young adults (age <45 years) [1-3] means that the young adult population will have an early start to a lifetime of metabolic complications including metabolic dysfunction-associated steatotic liver disease (MASLD) [4]. The global prevalence rates of MASLD are steadily increasing, with similar prevalence rates in young adults compared to adults ages 45 to 59 years [5]. An important step in curtailing the progression of MASLD to cirrhosis is early identification of clinically significant fibrosis or fibrosis stage ≥2 (F ≥ 2). The relevance of clinically significant fibrosis is that it is the precursor to cirrhosis, and when present, signals those at the highest risk of disease progression to cirrhosis [6]. Currently, the fibrosis-4 index (FIB-4) is simple to use and is associated with major adverse liver outcomes [7], so it is the most widely recommended first noninvasive step to risk stratify people with CMRFs for clinically significant fibrosis (F ≥ 2) [8-11]. MASLD is the fastest growing indication for liver transplant in young adults [12]. Identification of young adults with clinically significant fibrosis (F ≥ 2), which is still amenable to interventions, will facilitate earlier implementation of preventative strategies and treatment to reduce or halt the progression to cirrhosis.

The FIB-4 was developed in a cohort of patients with HIV/hepatitis C coinfection [13]. The thresholds were redefined to detect advanced fibrosis [defined as fibrosis stage 3 or more (F ≥ 3) based on the Nonalcoholic Steatohepatitis Clinical Research Network scoring system] in a cohort with biopsy-confirmed MASLD from hepatology clinics [14]. However, few young adults were included. Overall, the FIB-4 is considered less reliable in adults ≤35 years old and ≥65 years old [15, 16] as age is a variable included in the FIB-4 calculation. In addition, the accuracy of the currently used thresholds for the FIB-4 has been questioned in the setting of type 2 diabetes (T2D) [17] and for screening in primary care clinics [18]. Thus, the use of the FIB-4 for case detection of clinically significant fibrosis (F ≥ 2) in young adults needs to be redefined. Insulin resistance (IR) and CMRFs are increasingly common in young adults [19] and are associated with MASLD [20, 21]. We have previously shown that T2D and obesity together were positively associated with hepatic fibrosis in young adults [4]. As such, we concluded that CMRFs and/or a measurement of IR may improve risk stratification of young adults for clinically significant fibrosis (F ≥ 2).

The aim therefore was to determine if MASLD risk stratification can be improved by considering CMRFs and IR in young adults with and without T2D screened during routine outpatient clinic visits. We hypothesized that using CMRFs and/or a measurement of IR [homeostatic model assessment of insulin resistance (HOMA-IR)] would be superior to FIB-4 ≥ 1.3 alone in identifying young adults with clinically significant fibrosis (F ≥ 2) and would have better test characteristics in young adults compared to older adults.

## Materials and Methods

### Study Population

**Figure 1.**
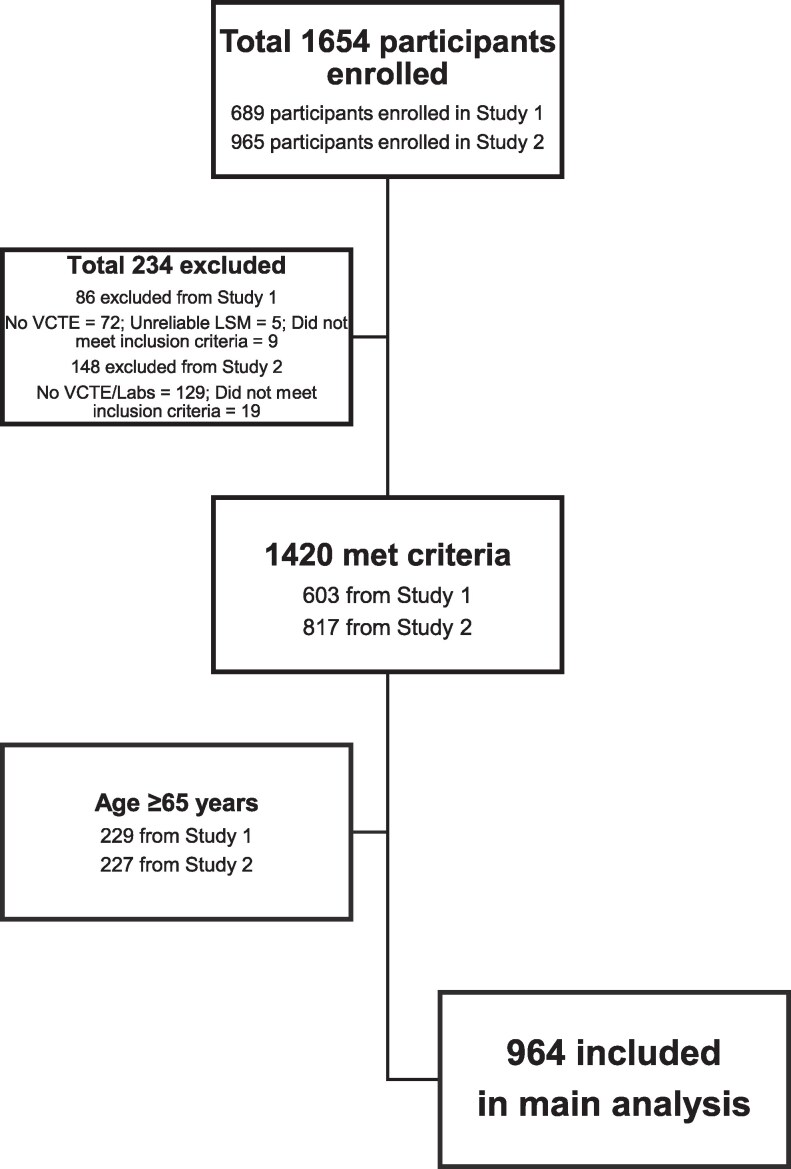
Consort diagram of study participants.

After obtaining approval by the institutional review board and informed consent from each participant, a total of 1654 participants were recruited from internal medicine, endocrinology, and family medicine outpatient clinics from 2 independent cross-sectional studies with 964 included in the final analysis (Fig. 1). Inclusion criteria and results for the first cohort (n = 603) were published previously [22]. Participants were adults (ages 21-79 years in the first cohort, 35-75 years in the second cohort) with or without a history of T2D with no known prior history of significant liver disease. We excluded participants who were 65 years or older (n = 456) as the specificity of the FIB-4 is low in this group [15]. Exclusion criteria in both cohorts were type 1 diabetes or any other form of diabetes not due to T2D; history of alcohol use of ≥20 g/day in women and ≥30 g/day in men (estimated by the Alcohol Use Disorders Identification Test questionnaire); a body mass index (BMI) ≥50 kg/m^2^; pregnancy or lactation, liver disease due to disorders other than MASLD (eg, alcohol-related liver disease, hepatitis B or C, autoimmune hepatitis, hemochromatosis, drug-induced hepatitis, etc.); medications that are associated with hepatic disease; and end-stage or decompensated heart, lung, liver, or kidney disease.

### Study Design

The 2 cohorts were cross-sectional studies, both to determine the prevalence of hepatic fibrosis (primary outcome) in people with or without T2D as measured by vibration-controlled transient elastography (VCTE) using FibroScan® (Echosens, Paris, France). We performed a complete medical history and physical examination in all participants. After fasting for 10 hours, blood samples were drawn for routine laboratory measurements, to assess IR, for the FIB-4 calculation [13], and to rule out other causes of hepatic disease. All participants were invited to be screened by VCTE for steatosis by the controlled attenuation parameter (CAP) and liver stiffness measurement (LSM). If the LSM by VCTE was ≥6.5 kPa, the participant was invited to undergo LSM by magnetic resonance elastography (MRE). A liver biopsy was offered if the VCTE-LSM was ≥8.2 kPa or MRE-LSM was ≥3.14 kPa.

#### Clinical and laboratory data

T2D was defined as a self-reported history, being on medication for T2D and/or a hemoglobin A1c (HbA1c) of ≥6.5%. Hypertension was defined as a self-reported history or use of antihypertensive therapy. Similarly, atherogenic dyslipidemia was defined as a self-reported history, on any lipid-lowering therapy, or abnormal lipid profile. Obesity was defined as a BMI ≥30 kg/m^2^ (BMI ≥25 kg/m^2^ for self-reported Asians). Laboratory studies included a complete blood count, complete metabolic panel, liver function test, HbA1c, lipid panel, plasma fasting insulin, free fatty acids (FFA), ferritin, viral hepatitis panel, and TSH. Plasma insulin was assessed with an ELISA kit from ALPCO (ALPCO Diagnostics Cat# 80-INSHU-E01.1, RRID:AB_2801438). Plasma FFA was assessed by an enzymatic colorimetric method from FUJIFULM Wako Chemicals diagnostics. The International Diabetes Federation criteria were used to define metabolic syndrome [23]. The FIB-4 was calculated as: FIB-4 = [age (years) × aspartate aminotransferase level (UI/L)]/(platelet count (10^9^/L) ×√[alanine aminotransferase level (U/L)] [13]. Participants with FIB-4 < 1.3 were considered as having a low risk of advanced liver fibrosis, between 1.3 to 2.67 was considered moderate or intermediate risk, and those with FIB-4 > 2.67 were classified as having a high risk of advanced liver fibrosis (F3 or F4). HOMA-IR was calculated with: [fasting insulin (μU/mL)] × fasting glucose (mg/dL)/405 [24]. Adipose tissue insulin resistance (Adipo-IR) was calculated with [fasting FFA (mmol/L)])×[fasting insulin (μU/mL)]. Those on exogenous insulin were excluded before calculating HOMA-IR and Adipo-IR.

#### VCTE-LSM

All participants underwent liver fibrosis screening by LSM with VCTE by experienced research staff who were manufacture-trained and certified in using the FibroScan device (Echosens model 530 with M and XL probes). In the supine position with the right arm abducted, the probe size recommended by the automatic probe selection tool was used to obtain a minimum of 10 measurements of the right liver lobe through an intercostal space at the same location. Hepatic steatosis was defined as a CAP ≥274 dB/m [25]. Hepatic fibrosis was assessed by the LSM and included in the final analysis only if valid (interquartile range/median ratio <30% and success rate exceeded 60%). Hepatic fibrosis was present if LSM ≥7.0 kPa and clinically significant fibrosis (≥F2) if LSM ≥8.0 kPa [9]. A participant was deemed higher risk for hepatic fibrosis when the LSM was ≥6.5 kPa, and further investigation with MRE was offered to confirm.

#### MRE

After detailed evaluation by a panel of clinical experts, participants with VCTE-LSM ≥6.5 kPa were invited to undergo MRE. MRE-LSM ≥2.65 kPa was defined as presence of any fibrosis or ≥F1, ≥3.14 kPa with clinically significant fibrosis, or ≥F2 and ≥3.53 kPa as advanced fibrosis or ≥F3 [26].

#### Liver biopsy

All participants with a high-risk picture for fibrosis or metabolic dysfunction-associated steatohepatitis (MASH) (VCTE-LSM ≥8.2 kPa or MRE-LSM of ≥2.65 kPa) were recommended to undergo an ultrasound-guided liver biopsy after an in-depth shared decision-making discussion of risks and benefits based on the participant's individual risk. The study pathologist (P.B.) evaluated all liver biopsies and was blinded to all clinical data. Clinical Research Network liver pathology criteria were used to determine the diagnosis of MASH and stage of fibrosis [27].

#### Establishing clinically significant fibrosis (F ≥ 2)

Clinically significant fibrosis (F ≥ 2) was defined as VCTE-LSM ≥8.0 kPa, LSM ≥3.14 kPa on MRE, and/or biopsy result consistent with fibrosis stage ≥2 [9, 26]. When a biopsy result was available, this was used to define the final diagnosis (n = 29) regardless of the MRE and VCTE results. If no biopsy was available (when participants refused or were unable to undergo biopsy), MRE-LSM (n = 25) was used over VCTE-LSM to define the diagnosis. If neither biopsy nor MRE were available, then VCTE (n = 819) was used for the final diagnosis. As not all participants underwent MRE or liver biopsy, the clinical characteristics of those who did have MRE or liver biopsy were compared to those who did not agree to undergo further investigation to ensure there were no clinically significant differences between the groups that could influence the results.

#### Statistical analysis

**Figure 2.**
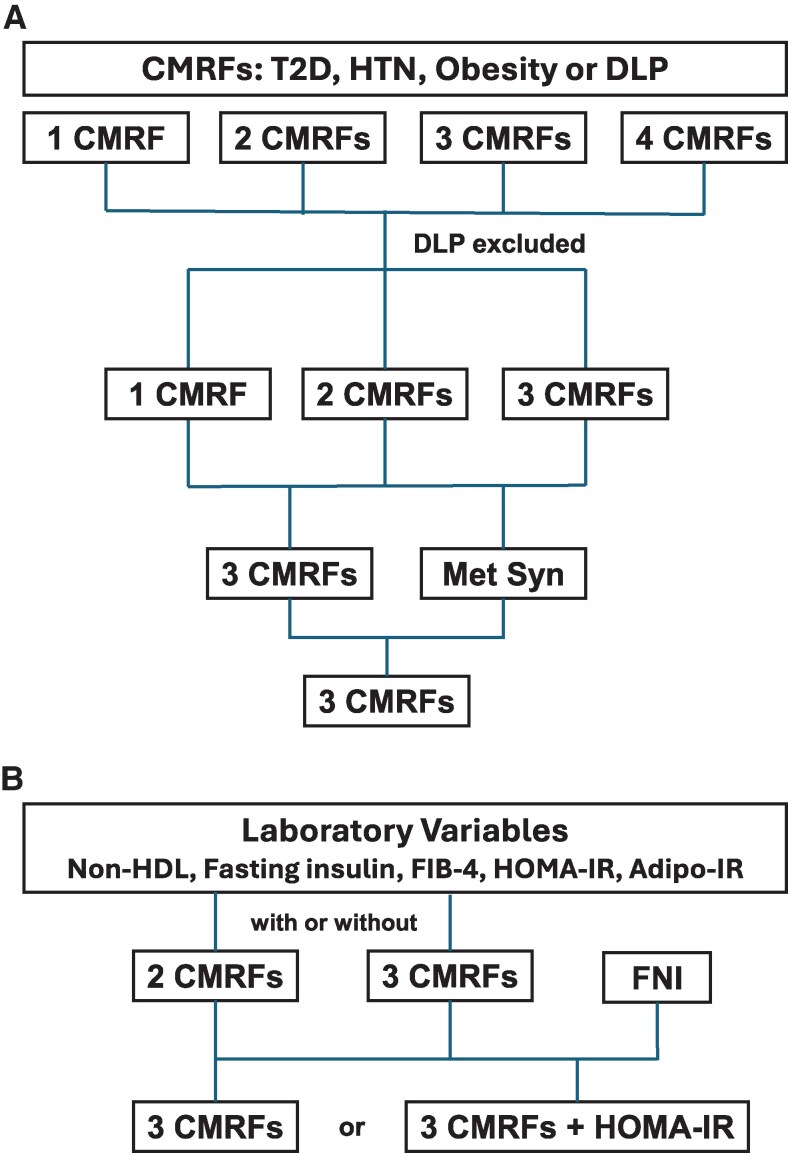
Flow chart depicting steps to determine the sensitivity and specificity of each variable and combination of variables: (A) clinical variables and (B) laboratory variables and noninvasive tests with and without cardiometabolic risk factors. Abbreviations: Adipo-IR, adipose tissue insulin resistance index; CMRFs, cardiometabolic risk factors; DLP, atherogenic dyslipidemia; FIB-4, fibrosis 4 index; FNI, fibrotic NASH index; HOMA-IR, homeostatic model assessment of insulin resistance; HTN, hypertension; Met Syn, metabolic syndrome; Non-HDL, non- high-density lipoprotein; T2D, type 2 diabetes.

Based on prior age definitions for young adults [4, 16, 28] and the lower reliability of the FIB-4 in older adults [15], the cohort was divided into 2 age groups a priori: <45 years and 45 to 64 years with data reported in this order. Data were summarized as percentages for categorical variables and mean (SD) or median (interquartile range) for continuous variables that are normally or not normally distributed, respectively. Categorical variables were analyzed with the Pearson χ^2^ or Fisher exact test. Continuous variables were analyzed with the Student's *t*-test when normally distributed and the Kruskal-Wallis test when not normally distributed. Missing data were handled by data removal. Differences with a significance level of *P* < .05 were considered statistically significant. Sensitivity, specificity, positive predictive value (PPV) and negative predictive value (NPV) for each variable and combination of variables were calculated (Fig. 2) in both age groups. Analyses were performed using JMP Pro 18.0.

## Results

### Participant Characteristics

Of the 964 included in the final analysis, 25% were <45 years (young adults) and 75% were 45 to 64 years (Table 1). As expected, the prevalence of metabolic syndrome was lower in young adults (age <45 yrs: 56% vs age 45-64 yrs: 73%, *P* < .001). In those with T2D, mean HbA1_c_ was slightly higher in young adults despite no differences in the use of medications for T2D. Mean aspartate aminotransferase and alanine aminotransferase concentrations were no different. Young adults had mildly higher low-density lipoprotein and non-high-density lipoprotein (HDL) concentrations, as well as slightly lower HDL cholesterol. When analyzed by statin therapy use, however, this was reversed in those not on statin therapy; ie, young adults not on statins had slightly lower low-density lipoprotein and non-HDL concentrations compared to older adults not on statins. There were no differences in markers of IR (HOMA-IR or Adipo-IR). In addition, there were no differences in the prevalence of hepatic steatosis (CAP ≥274 dB/m 57% vs 61%; *P* = .292), hepatic fibrosis (9% vs 13%; *P* = .09), or clinically significant fibrosis (F ≥ 2) (7% vs 9%; *P* = .29), although there was a trend to be lower in young adults (Table 1). There was no difference in the frequency of clinically significant fibrosis in those who used and did not use glucagon-like peptide-1 receptor agonists (GLP-1RA) (≥F2: 17% on GLP-1RA vs 11% not on GLP-1RA, *P* = .22).

**Table 1.** Participant characteristics

Clinical parameters	Age <45 years (n = 243)	Age 45-64 years (n = 721)
Age (years)*^a^*	39 (4)	56 (6)
Female sex (%)	56	58
Race (%)		
White	61	65
Black	23	23
Asian	10	7
Other	6	5
Hispanic ethnicity (%)	11	7
BMI (kg/m^2^)	33 (7)	32 (6)
BMI categories (%)		
Overweight	19	26
Obesity	67	63
T2D (%)	41	65
On medication	93	90
Hypertension	61	80
Dyslipidemia	80	91
Metabolic syndrome	56	73
Laboratory data		
HbA1c	6.3 (1.9)	6.7 (1.7)
Without T2D	5.3 (0.4)	5.5 (0.4)
With T2D	7.9 (2.2)	7.4 (1.7)
AST (U/L)	23 (13)	23 (10)
ALT (U/L)	25 (20)	24 (17)
Total cholesterol (mg/dL)	180 (40)	176 (51)
Triglycerides (mg/dL)	142 (107)	140 (84)
HDL-C (mg/dL)	47 (13)	49 (14)
LDL-C (mg/dL)	107 (37)	98 (41)
Non-HDL-C (mg/dL)	133 (39)	125 (43)
Fasting insulin (μU/mL)	12 (8)	12 (6)
FFA (mmol/L)	0.31 (0.14)	0.33 (0.17)
Adiponectin (μg/mL)	5.2 (3.2)	5.6 (3.6)
Adipo-IR (mmol/L × μU/mL)	4.8 (4.1)	4.4 (3.8)
HOMA-IR (μU/mL × mg/dL)	3.8 (3.5)	3.6 (2.7)
Presence of hepatic steatosis or fibrosis (%)		
Hepatic steatosis by CAP	57	61
Any fibrosis (≥ F1)	9	14
Clinically significant fibrosis (≥F2)	7	9

Results are mean and SD.

Abbreviations: Adipo-IR, adipose tissue insulin resistance index; ALT, alanine aminotransferase; AST, aspartate aminotransferase; BMI, body mass index; CAP, liver steatosis measured by controlled attenuation parameter; clinically significant fibrosis (≥F2)—people with liver steatosis and liver fibrosis measured either by vibration-controlled transient elastography and/or magnetic resonance elastography and/or liver biopsy.; FFA, free fatty acids; HbA1c, hemoglobin A1c; HDL-C, high-density lipoprotein cholesterol; HOMA-IR, homeostatic model assessment of insulin resistance; LDL-C, low-density lipoprotein cholesterol; T2D, type 2 diabetes mellitus.

^a^Interquartile range: age < 45 years: 36-42 years (range: 21-44 years); 45-64 years: 51-61 years (range: 45-64 years).

### Performance of Clinical Variables for the Diagnosis of Clinically Significant Fibrosis (F ≥ 2)

To assess the impact of using CMRFs or HOMA-IR to improve the performance of the FIB-4 for categorizing participants at risk of having MASLD with clinically significant fibrosis, we first determined the test characteristics (sensitivity, specificity, PPV, and NPV) for the presence of different numbers of CMRFs for the prespecified age groups (Table S1) [29]. The presence of T2D, hypertension (HTN), and obesity together yielded the best sensitivity (75%) and specificity (71%), which was better than utilizing at least 1 or 2 of these risk factors alone. Atherogenic dyslipidemia and non-HDL concentrations mostly occurred with other CMRFs (90% of young adults with atherogenic dyslipidemia had at least 1 CMRF; 97% of young adults with non-HDL concentration >130 mg/dL had at least 1 CMRF); thus they did not offer any additional discriminatory power compared to using T2D, HTN, and obesity alone (Tables S1 and S2) [29].

### Performance of Clinical Variables for the Diagnosis of Clinically Significant Fibrosis (F ≥ 2) in Young Adults

We then calculated the test characteristics for the FIB-4, CMRFs, the HOMA-IR, and various combinations of these risk factors in young adults (Table 2; Table S2 for ages 45-64 years) [29]. The FIB-4 had a very low sensitivity (15%) but high specificity (97%). While the HOMA-IR had 100% sensitivity, its specificity was only 57%. The presence of 2 CMRFs had a similar sensitivity to 3 CMRFs (75% vs 75%) but lower specificity (61% vs 71%) with the PPV influenced by the low prevalence rate in young adults with 2 CMRFs (only 65 young adults had 2 CMRFs present with a prevalence of clinically significant fibrosis of only 2.4% in young adults with 0-2 CMRFs). As such, we focused on 3 CMRFS. The presence of 3 CMRFs had a good balance of both sensitivity (75%) and specificity (71%) (Table S1; Fig. S1A) [29]. When 3 CMRFs were combined with the FIB-4, there was a significant loss in sensitivity (15% with FIB-4% to 8% with 3 CMRFs with FIB-4). Combining CMRFs with the HOMA-IR proved to be very useful. Both sensitivity and specificity improved when the HOMA-IR was added to CMRFs. Using 3 CMRFs with the HOMA-IR (Table 2; Fig. S1B) [29] had the highest combined sensitivity and specificity (sensitivity 78% and specificity 81%). In a subgroup analysis in only those where the HOMA-IR was available, the results were unchanged. We also analyzed the entire cohort by sex and diabetes status to determine the effect of each factor on test characteristics (Table S3) [29].

**Table 2.** Test characteristics for cardiometabolic risk factors and laboratory values to predict clinically significant fibrosis (≥F2) in young adults (age <45 years) and older adults (age 45-64 years)

Risk factor	Definition	Sensitivity	Specificity	PPV	NPV
<45 years					
3 CMRFs	Presence of 3 CMRFs together (T2D, HTN + obesity)	75 (48-93)	71 (64-76)	15 (11-20)	98 (95-99)
FIB-4	≥1.3	15 (2-46)	97 (94-99)	25 (7-60)	95 (94-96)
2 CMRFs + FIB-4	Presence of only 2 CMRFs (T2D, HTN, or obesity) and FIB-4 ≥ 1.3	50 (1-99)	99 (95-100)	33 (7-78)	99 (97-100)
3 CMRFs + FIB-4	Presence of 3 CMRFs together (T2D, HTN + obesity) and FIB-4 ≥ 1.3	8 (0.2-36)	100 (98-100)	100 (3-100)	94 (93-95)
HOMA-IR	≥3.0	100 (66-100)	57 (49-65)	11 (10-13)	100 (96-100)
2 CMRFs + HOMA-IR*^a^*	Presence of only 2 CMRFs (T2D, HTN, or obesity) and HOMA-IR ≥3.0	100 (16-100)	82 (74-88)	8 (6-11)	100 (97-100)
3 CMRFs + HOMA-IR*^a^*	Presence of 3 CMRFs together (T2D, HTN + obesity) and HOMA-IR ≥3.0	78 (40-97)	81 (75-87)	18 (12-27)	98 (95-100)
45-64 years					
3 CMRFs	Presence of 3 CMRFs together (T2D, HTN + obesity)	79 (67-89)	55 (51-59)	15 (13-16)	97 (95-98)
FIB-4	≥1.3	42 (28-58)	72 (68-76)	10 (7-14)	94 (93-96)
2 CMRFs + FIB-4	Presence of only 2 CMRFs (T2D, HTN, or obesity) and FIB-4 ≥ 1.3	63 (25-92)	85 (81-89)	9 (5-16)	99 (97-100)
3 CMRFs + FIB-4	Presence of 3 CMRFs together (T2D, HTN + obesity) and FIB-4 ≥ 1.3	31 (18-47)	89 (86-91)	17 (11-25)	95 (93-95)
HOMA-IR	≥3.0	100 (86-100)	55 (50-60)	11 (10-12)	100 (99-100)
2 CMRFs + HOMA-IR*^a^*	Presence of only 2 CMRFs (T2D, HTN, or obesity) and HOMA-IR ≥3.0	88 (47-100)	78 (73-83)	11 (8-14)	100 (97-100)
3 CMRFs + HOMA-IR*^a^*	Presence of 3 CMRFs together (T2D, HTN + obesity) and HOMA-IR ≥3.0	68 (47-85)	73 (69-77)	12 (9-16)	98 (96-99)

Test characteristics reported as the value (95% confidence interval).

Abbreviations: CMRFs, cardiometabolic risk factors (type 2 diabetes, hypertension, obesity); FIB-4, fibrosis-4 index; FN, false negative; FP, false positive; HOMA-IR, homeostatic model assessment of insulin resistance; HTN, hypertension; NPV, negative predictive value; PPV, positive predictive value; T2D, type 2 diabetes; TN, true negative; TP, true positive.

^a^HOMA-IR ≥3.0 μU/mL × mg/dL.

### Using the FIB-4 in Young Adults with T2D or Obesity

We examined the utility of the FIB-4 in young adults with T2D. Unfortunately, if the FIB-4 was used in young adults with T2D to identify clinically significant fibrosis, the sensitivity was only 10%; thus 90% of cases would be missed (false negatives = 9, true positive = 1). If the FIB-4 was used in all young adults with obesity and at least 1 other risk factor (T2D, HTN, hypertriglyceridemia, or low HDL per International Diabetes Federation criteria), then the sensitivity would only be 15% with 85% of cases missed. However, if we decreased the FIB-4 threshold to >0.81 (based on the Youden's index for young adults), the sensitivity increases to 60% with a specificity of 84% in young adults with T2D, and the sensitivity increases to 69% with a specificity of 77% in young adults with obesity.

### Impact of Overweight and Obesity in Clinically Significant Fibrosis

**Figure 3.**
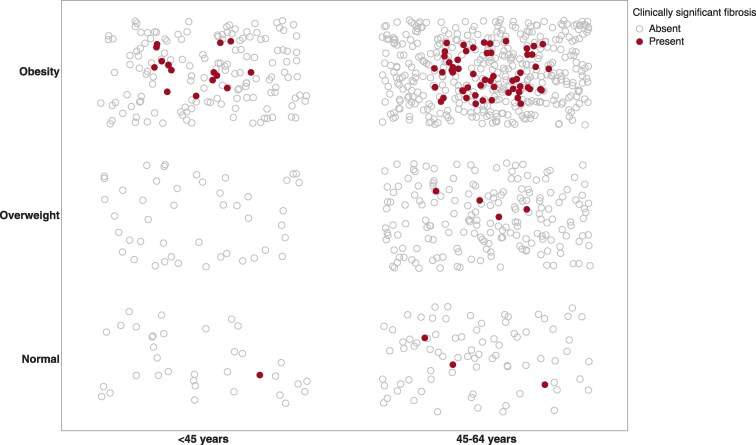
Frequency of clinically significant fibrosis by age group and weight. *P* < .01 when stratified by age group.

In young adults, overweight had no impact on the development of clinically significant fibrosis (Fig. 3). A similar finding was noted for adults ages 45 to 64 years. Markers of IR were not significantly different in young adults with an overweight BMI compared to normal BMI (Fig. S2; HOMA-IR: 1.6 ± 1.1 vs 2.8 ± 3.3 μU/mL × mg/dL, *P* = .22; Adipo-IR: 2.3 ± 1.9 vs 3.5 ± 2.2 mmol/L × μU/mL, *P* = .27) [29]. Compared to young adults with a normal BMI, young adults with obesity had triple the HOMA-IR (1.6 ± 1.1 vs 5.0 ± 4.5 μU/mL × mg/dL, *P* < .01) and double the Adipo-IR (2.3 ± 1.9 vs 5.8 ± 4.6 mmol/L × μU/mL, *P* < .01). Interestingly, a similar pattern was seen in the 45 to 64 years age group.

### Comparison of Participants with and Without MRE or Liver Biopsy

To validate our VCTE-LSM prevalence findings and risk-stratification strategy, clinical/metabolic parameters were not significantly different between the participants with clinically significant fibrosis who underwent MRE or liver biopsy when compared to those who did not except for the presence of metabolic syndrome, which was more frequent in participants who underwent MRE or liver biopsy (Table 3).

**Table 3.** Patient characteristics of patients with clinically significant liver fibrosis by VCTE (VCTE-LSM ≥8.0 kPa) that underwent magnetic resonance elastography or liver biopsy vs those who did not

Clinical parameters	Patients with kPa ≥8.0 and MRE/Bx (n = 34)	Patients with kPa ≥8.0 without MRE/Bx (n = 48)	*P*-value
Age (years)	54 ± 9	53 ± 9	.63
Age <45 years (%)	18	19	.90
Female sex (%)	50	48	.85
BMI (kg/m^2^)	38 ± 6	38 ± 6	.98
BMI (%)			.05
Overweight	12	2	
Obese	88	90	
T2D (%)	88	85	.71
Hypertension (%)	94	92	.68
Dyslipidemia (%)	97	92	.32
Coronary artery disease (%)	18	6	.10
Metabolic syndrome (%)	100	83	.01
HbA1c (%)	7.5 ± 1.6	7.7 ± 2.0	.63
AST (U/L)	34 ± 14	31 ± 26	.51
ALT (U/L)	44 ± 25	37 ± 36	.42
Total cholesterol (mg/dL)	183 ± 97	169 ± 49	.46
TG (mg/dL)	205 ± 135	168 ± 96	.22
HDL-C (mg/dL)	45 ± 13	41 ± 9	.22
LDL-C (mg/dL)	84 ± 49	92 ± 33	.46
CAP (dB/m)	353 ± 31	343 ± 52	.26
LSM (kPa)	11.4 ± 3.6	11.5 ± 4.1	.95

Abbreviations: ALT, alanine aminotransferase; AST, aspartate aminotransferase; BMI, body mass index; CAP, controlled attenuation parameter; HbA1c, hemoglobin A1c; HDL-C, high-density lipoprotein cholesterol; LDL-C, low-density lipoprotein cholesterol; LSM, liver stiffness measurement; MRE, magnetic resonance elastography; T2D, type 2 diabetes mellitus; TG, triglycerides; total chol, total cholesterol; VCTE, vibration controlled transient elastography.

## Discussion

Given the increasing prevalence of MASLD in young adults [4, 5], easy identification of those with clinically significant fibrosis (F ≥ 2) is needed to prevent progression to cirrhosis. The FIB-4 is the recommended screening tool to identify hepatic fibrosis but is known to underperform in young adults [15]. As such, we aimed to identify how to improve its diagnostic performance using readily available clinical (ie, CMRFs) and metabolic (ie, IR measured by the HOMA-IR) variables to better identify young adults who should undergo further screening for clinically significant fibrosis (F ≥ 2). We show that considering the presence of CMRFs with the HOMA-IR had the best overall performance and can potentially be used to improve risk stratification in selected young adults. This study has a significant clinical implication as it establishes that in young adults with 3 CMRFs (T2D, obesity, HTN) clinicians should further evaluate with imaging (transient elastography) or consider referral to a specialist for clinically significant fibrosis (F ≥ 2), independent of the FIB-4. Moreover, it establishes that when young adults have IR with 3 CMRFs, their chance of having clinically significant fibrosis (and eventually cirrhosis) increases considerably and to a degree much higher than previously appreciated. Taken together, if confirmed in larger studies, our findings can improve current diagnostic strategies and may modify current screening guidelines for risk-stratifying young adults at risk of MASLD with moderate to advanced fibrosis.

The FIB-4 is recommended by all recent guidelines as the initial noninvasive screen for hepatic fibrosis [8-11]. Guideline-based recommendations propose to use the FIB-4 to risk-stratify all adults with T2D [10] and those with obesity and additional CMRFs [8-11]. The rationale for using the FIB-4 is its simplicity, low cost, and easily available variables required for calculation [7]. Unfortunately, the FIB-4 includes age in its calculation, leading to lower accuracy in younger and older adults [15]. We show that CMRFs (T2D, HTN, and/or obesity) had better sensitivity with similar NPV compared to the FIB-4. Kim et al [30] compared the performance of the FIB-4 to the aminotransferase-to-platelet ratio and nonalcoholic fatty liver disease fibrosis score to identify advanced fibrosis (≥F3) on MRE in a community setting. Like our study, they show reduced performance of the FIB-4 in young adults (<45 years) [30]. In contrast, Vali et al showed no significant differences in the FIB-4 area under the curve by age groups to identify advanced fibrosis on liver biopsy in the LITMUS Metacohort, which is enriched with MASLD [28]. Both studies, however, focused on identifying advanced fibrosis (≥F3), whereas our goal was to identify clinically significant fibrosis (≥F2), which would facilitate earlier implementation of lifestyle and pharmacological intervention to avert cirrhosis.

Insulin resistance plays a pivotal role in the development of MASLD [21, 31]. Markers of IR (like HOMA-IR) should theoretically aid the identification of those at highest risk for “at-risk” MASH or clinically significant fibrosis (F ≥ 2). The HOMA-IR has been shown to have an acceptable correlation with more invasive measures of insulin sensitivity (euglycemic insulin clamp or IV glucose tolerance test) [24]. The threshold required to identify people with clinically significant fibrosis (F ≥ 2), however, is influenced by the population studied. A HOMA-IR threshold of ≥2.5 was previously shown to have a sensitivity of 72% and specificity of 94% in a Brazilian cohort to detect MASLD [32]. In a Japanese cohort after multivariate analysis, a HOMA-IR of ≥2.90 significantly increased the risk of biopsy-proven hepatic fibrosis [33]. In a NHANES study using VCTE to diagnose hepatic fibrosis, A HOMA-IR ≥3.11 increased the risk of hepatic fibrosis with a sensitivity of 60%, specificity of 71%, and area under the curve of 0.68 [34]. In another NHANES study, up to 40% of young adults without T2D had a HOMA-IR of ≥2.5 [19]. As obesity and T2D are known factors that increase HOMA-IR, the discriminatory power of the HOMA-IR ≥2.5 to identify those with clinically significant fibrosis (F ≥ 2) will be lower in a cohort enriched with metabolic disease. In our cohort, 55% of young adults met the criteria for metabolic syndrome and 65% had obesity. The optimal threshold for the HOMA-IR in our cohort was 3.1 (Fig. S3) [29]. We therefore opted for a higher HOMA-IR threshold of ≥3.0 to improve specificity [32]. As such, the HOMA-IR increased both the sensitivity and specificity of CMRFs. Broad implementation of the HOMA-IR across populations, though, is problematic. As race/ethnicity, presence of T2D, use of exogenous insulin, and the interassay variation of insulin measurements all influence the HOMA-IR, and due to the fact that serum insulin concentration is not routinely measured in the clinic, population-based or at least local thresholds will need to be established before broadly recommending the use of the HOMA-IR across all clinical settings. However, the HOMA-IR may have better discriminatory power in those without CMRFs as it would allow easy identification of IR, a hallmark pathophysiological step in the development of hepatic steatosis and fibrosis.

Interestingly, in our cohort the diagnostic performance of the FIB-4 in those ages 45 to 64 years was lower than expected. In this group, 65% had a history of T2D, and some studies have shown that a lower threshold for the FIB-4 may be needed to identify clinically significant fibrosis (≥F2) in people with T2D [17]. In addition, the prevalence of advanced fibrosis was low (5% with LSM ≥9.7 kPa), which would affect the PPV of the FIB-4 in our cohort. Caviglia et al [35] assessed the use of a 2-tier screening approach with the FIB-4 followed by VCTE in people with T2D. This improved diagnostic performance as it reclassified 70% of FIB-4 ≥ 1.3 as they had a VCTE <8.0 kPa. Like our cohort, however, the sensitivity and specificity of FIB-4 ≥ 1.3 was underwhelming, and their study was comparable to our findings (sensitivity 43% and specificity 82%). As such, the FIB-4 threshold likely would need to be adjusted based on the outcome desired (≥F2 vs ≥F3) and the population being studied (age, T2D vs no T2D, hepatology clinic vs nonhepatology clinic).

Overweight is thought to carry an elevated risk for MASLD. Overweight was therefore listed as 1 of the CMRFs to fulfill the criteria for defining MASLD [11]. We therefore sought to assess the risk of clinically significant fibrosis (F ≥ 2) in our cohort by BMI category (Fig. 3). Surprisingly, 63% of young adults with an overweight BMI had at least 1 other CMRF. Despite this, there were no cases of clinically significant fibrosis in young adults with an overweight BMI. Low rates of clinically significant fibrosis were also seen in the older age group despite 86% in the 45- to 64-year-old group who had an overweight BMI also had at least 1 other CMRF. Overweight therefore is a weak criterion to screen on its own or with even another CMRF. It appears, however, that obesity potentiates the development of clinically significant fibrosis in the setting of other CMRFs [4]. As visceral adiposity increases, the risk of MASH and advanced fibrosis increases [36]. Increased visceral adiposity decreases adiponectin [37], a known marker of adipose tissue health, leading to both hepatic and adipose tissue IR, which are significant precursors for hepatic fibrosis in MASLD [37]. In keeping with this, IR markers were significantly higher in adults with obesity compared to those with normal and overweight BMI across all age groups in our cohort and significantly higher in those with clinically significant fibrosis compared to those without clinically significant fibrosis. As such, Adipo-IR could be a potential precision medicine tool clinicians can use in the future to risk stratify patients with lean and overweight BMIs as it not only identifies a higher risk group but also has been associated with treatment response to pioglitazone [38, 39].

The clinical characteristics of our participants were representative of the usual patients attending an outpatient internal medicine, endocrinology, or family medicine clinic. Being in the Southeast United States, where obesity prevalence is higher than other regions [40], may have biased our cohort to have a higher frequency of metabolic disease. Despite this, the overall frequency of clinically significant fibrosis was low and consistent with other recent prevalence studies using VCTE-LSM in participants with and without T2D [35, 41]. Assessing simultaneously CMRFs and IR gives additional strength to our study, in addition to calculating the FIB-4 and transient elastography testing. Another strength is that among young adults with clinically significant fibrosis, as many as 40% underwent MRE and/or biopsy for confirmation, something not done before in young adults attending outpatient medical clinics. However, our findings will need to be replicated in other cohorts to determine applicability across different populations. In addition, the HOMA-IR is currently not a routine clinical variable. A larger study with the aim to establish baseline population thresholds utilizing a standardized insulin assay is required before we can broadly apply the assessment of IR by the HOMA-IR in the clinic.

In summary, FIB-4 ≥ 1.3 has poor sensitivity to use as a screening tool for clinically significant fibrosis (F ≥ 2) in young adults. Using the presence of CMRFs (ie, T2D, HTN, and obesity) may be an alternative to help identify high-risk individuals in this population. Performance may be improved by selectively adding the HOMA-IR using clinical judgement. This can be implemented in clinical practice by standardizing the normal insulin range for local/regional testing to facilitate early identification of clinically significant fibrosis or MASH in selected individuals. Further research is needed to improve screening strategies for young adults at high risk of clinically significant fibrosis to prevent future cirrhosis.

## Data Availability

Some or all datasets generated during and/or analyzed during the current study are not publicly available but are available from the corresponding author on reasonable request.
